# Effect of Latin dance on physical and mental health: a systematic review

**DOI:** 10.1186/s12889-023-16221-6

**Published:** 2023-07-11

**Authors:** Xutao Liu, Kim Geok Soh, Roxana Dev Omar Dev

**Affiliations:** Department of Sports Studies, Faculty of Educational Studies, Universiti Putra Malaysia, Serdang, Malaysia

**Keywords:** Latin dance_1_, Physical activity_2_, Physical health_3_, Mental health_4_, Exercise intervention_5_, Public health_6_

## Abstract

**Background:**

Latin dance is a well-liked physical activity. It has gained increasing attention as an exercise intervention for improving physical and mental health outcomes. This systematic review examines the effects of Latin dance on physical and mental health.

**Methods:**

Preferred Reporting Items for Systematic Reviews and Meta-analysis (PRISMA) were used to report the data for this review. To gather research from the literature, we used recognized academic and scientific databases such SportsDiscus with Full Text, PsycINFO, Cochrane, Scopus, PubMed, and Web of Science. The systematic review only included 22 studies out of the 1,463 that matched all inclusion criteria. The PEDro scale was used to rate each study’s quality. 22 research received scores between 3 and 7.

**Results:**

Latin dance has been demonstrated to promote physical health by helping people lose weight, improve cardiovascular health, increase muscle strength and tone, and improve flexibility and balance. Furthermore, Latin dance can benefit mental health by reducing stress, improving mood, social connection, and cognitive function.

**Conclusions:**

Finding from this systematic review provide substantial evidence that Latin dance has effect on physical and mental health. Latin dance has the potential to be a powerful and pleasurable public health intervention.

**Systematic Review Registration:**

CRD42023387851, https://www.crd.york.ac.uk/prospero.

## Introduction

Physical activity has long been recognized as a fundamental component of health promotion [1]. Regular physical activity has been associated with a reduced risk of chronic diseases, such as cardiovascular disease, diabetes, and various types of cancer [2, 3]. It also contributes to maintaining a healthy body weight [4], improving bone density [5], and muscular strength [6], as well as enhancing mental well-being [7]. Despite these benefits, many individuals encounter challenges in adhering to a regular exercise routine, often due to barriers such as time constraints, lack of motivation, or limited access to facilities.

In this context, dance has emerged as a promising form of physical activity that can promote engagement and improve health outcomes [8]. Latin dance, originating from Latin America, encompasses various sports dances such as rumba, samba, Cha Cha Cha, bullfighting dances, and cowboy dances [9, 10]. It has gained increasing attention in recent years regarding its potential health benefits. Latin dance steps are characterized by flexibility, with the dancer’s body amplifying power, speed, and body posture in synchronization with the rhythm of the music, showcasing the beauty of the human body [11]. Latin dance provides a unique component to aerobic training: interpersonal communication and involvement [12].

The aim of this systematic review is to examine the effect of Latin dance on physical and mental health. Specifically, we will explore the impact of Latin dance on cardiovascular endurance, muscular strength, flexibility, balance, anxiety, depression, mood, self-esteem, social connection, and cognitive function. Additionally, we will examine the potential benefits of Latin dance as a form of exercise and stress relief. By adopting a systematic review methodology, we can identify, select, and critically appraise relevant literature to provide a comprehensive analysis of the current state of knowledge. Our review will encompass studies published in English, involving diverse populations such as young adults, older adults, and individuals with specific health conditions [13, 14]. The findings from this systematic review will offer valuable insights into the potential health benefits of Latin dance and inform future research and practice. Moreover, understanding the physical and mental health advantages of Latin dance [15] can provide essential information for healthcare professionals, fitness instructors, and individuals seeking alternative forms of exercise and stress relief.

From a physiological perspective, exercise intervention programs are commonly employed to promote physical activity and improve health outcomes. Latin dance presents an appealing and enjoyable form of exercise, particularly for individuals who are less inclined toward traditional exercise methods. Extensive research has demonstrated the wide-ranging physical benefits of Latin dance, including increased strength and flexibility in various muscle groups such as the abdomen, back, gluteal region, and upper extremities [16–18]. Latin dance involves whole-body movements that enhance the functionality of different body parts, particularly the muscles and bones of the lower limbs, contributing to overall health [19]. The rhythmic nature of Latin dance allows for freedom of movement and smooth transitions [20], facilitating both physical and mental well-being [21]. Regular participation in Latin dance practice can positively influence proper sitting and standing posture [19, 22], while dancers often exhibit better control over their physique, maintaining a healthy appearance through regular exercise [23]. Furthermore, Latin dance can improve body flexibility, reducing the risk of joint injuries by promoting flexible muscles and expanded range of motion [9]. Participation in Latin dance has also been found to benefit cardiovascular health, with research indicating that engaging in Latin dance at least twice a week can reduce the risk of heart attack-related mortality by 30 to 50% [10]. It is worth noting that adequate sleep and a balanced diet are essential for sustaining energy during Latin dance practice, as physical activity often involves significant exertion and perspiration, which can lead to nutritional deficiencies [24]. Therefore, Latin dance serves a protective function in terms of health.

In relation to mental health, it is a crucial component of overall well-being. Regular physical activity has been shown to have positive effects on stress reduction, anxiety alleviation, and depression mitigation [25]. Dance, with its social and creative aspects, can offer additional benefits. Latin dance, as a social dance form, has the potential to enhance social connectedness and improve mood. For instance, a 12-week Latin dance program was found to enhance mood and quality of life in older adults with mild cognitive impairment [26], while another study reported a reduction in symptoms of anxiety and depression in women with fibromyalgia following a 12-week Latin dance program [27]. Learning Latin dance can foster self-confidence, optimism, mutual understanding, and cooperation. Despite Latin dance being recognized as an effective form of exercise, limited research has investigated its impact on physical and mental health. Through a comprehensive examination of the existing literature, this review aims to provide a comprehensive assessment of the effects of Latin dance on physical and mental health and highlight its potential as an effective and enjoyable public health intervention.

## Materials and methods

### Protocol and registration

This review was collected, selected and analyzed in accordance with the guidelines for Systematic Reviews and Meta-Analyses (PRISMA 2020) guidelines [28], and this study was entered in the International Prospective Register of Systematic Reviews; https://www.crd.york.ac.uk/prospero, CRD42023387851.

### Search strategy

The search for literature was conducted in six international databases: EBSCOhost-SportsDiscus with Full Text, APA PsycINFO, Cochrane, Scopus, PubMed, and Web of Science. The search took place on June 23, 2023. In each database, a search was conducted by title, taking a predefined combination of keywords: (“Latin dance” OR “dance” OR “sports dance” OR “tango” OR “rumba” OR “cha cha cha” OR “samba” OR “bullfighting dances” OR “cowboy dances”) AND (“physical” OR “physical health” OR “physical activity” OR “physical fitness ” OR “bodily health” OR “physiological well-being” OR “somatic health” OR “physical well-being” OR “overall health” OR “bodily fitness”) AND (“Mental Health” OR “psychology” OR “psychological well-being” OR “emotional well-being” OR “mental well-being” OR “psychological health” OR “emotional health” OR “mental well-being” OR “psychological disorders”). Furthermore, we examined other similar papers in the reference lists of the research included in the review, as well as the reference lists of prior relevant reviews. All titles have been manually checked for inclusion. For further relevant citations, reference lists of retrieved publications, author names, and review articles were manually retrieved. Table 1 shows the search terms in the database. Table 2 shows the entire search technique for all databases.

**Table 1:** Search terms in the database

“Latin dance”	Operator	“Physical”	Operator	“Mental health”
“Latin dance” OR “dance” OR “sports dance” OR “tango” OR “rumba” OR “cha cha cha” OR “samba” OR “bullfighting dances” OR “cowboy dances”	AND	“physical” OR “physical health” OR “physical activity” OR “physical fitness ” OR “bodily health” OR “physiological well-being” OR “somatic health” OR “physical well-being” OR “overall health” OR “bodily fitness”	AND	“Mental Health” OR “psychology” OR “psychological well-being” OR “emotional well-being” OR “mental well-being” OR “psychological health” OR “emotional health” OR “mental well-being” OR “psychological disorders”

**Table 2:** The total number of hits for the entire search strategy for the databases

Database	Complete Search Strategy	Results
SportsDiscus with Full Text (1969–2023)	SU (“Latin dance” OR “dance” OR “sports dance” OR “tango” OR “rumba” OR “cha cha cha” OR “samba” OR “bullfighting dances” OR “cowboy dances” ) AND SU ( “physical” OR “physical health” OR “physical activity” OR “physical fitness ” OR “bodily health” OR “physiological well-being” OR “somatic health” OR “physical well-being” OR “overall health” OR “bodily fitness” ) AND SU ( “Mental Health” OR “psychology” OR “psychological well-being” OR “emotional well-being” OR “mental well-being” OR “psychological health” OR “emotional health” OR “mental well-being” OR “psychological disorders”)	120
APA PsycINFO (1986–2023)	SU ( “Latin dance” OR “dance” OR “sports dance” OR “tango” OR “rumba” OR “cha cha cha” OR “samba” OR “bullfighting dances” OR “cowboy dances” ) AND SU ( “physical” OR “physical health” OR “physical activity” OR “physical fitness ” OR “bodily health” OR “physiological well-being” OR “somatic health” OR “physical well-being” OR “overall health” OR “bodily fitness” ) AND TX ( “Mental Health” OR “psychology” OR “psychological well-being” OR “emotional well-being” OR “mental well-being” OR “psychological health” OR “emotional health” OR “mental well-being” OR “psychological disorders”	174
Cochrane (2013–2023)	Keyword: “Latin dance” OR “dance” OR “sports dance” OR “tango” OR “rumba” OR “cha cha cha” OR “samba” OR “bullfighting dances” OR “cowboy dances” AND “physical” OR “physical health” OR “physical activity” OR “physical fitness ” OR “bodily health” OR “physiological well-being” OR “somatic health” OR “physical well-being” OR “overall health” OR “bodily fitness” AND “Mental Health” OR “psychology” OR “psychological well-being” OR “emotional well-being” OR “mental well-being” OR “psychological health” OR “emotional health” OR “mental well-being” OR “psychological disorders” in Keyword	350
Scopus (2013–2023)	TITLE-ABS-KEY ( “Latin dance” OR “dance” OR “sports dance” OR “tango” OR “rumba” OR “cha cha cha” OR “samba” OR “bullfighting dances” OR “cowboy dances” AND “physical” OR “physical health” OR “physical activity” OR “physical fitness " OR “bodily health” OR “physiological well-being” OR “somatic health” OR “physical well-being” OR “overall health” OR “bodily fitness” AND “Mental Health” OR “psychology” OR “psychological well-being” OR “emotional well-being” OR “mental well-being” OR “psychological health” OR “emotional health” OR “mental well-being” OR “psychological disorders” )	469
PubMed (1989–2023)	(“Latin dance“[Title/Abstract] OR “dance“[Title/Abstract] OR “sports dance“[Title/Abstract] OR “tango“[Title/Abstract] OR “rumba“[Title/Abstract] OR “cha cha cha“[Title/Abstract] OR “samba“[Title/Abstract] OR “bullfighting dances“[Title/Abstract] OR “cowboy dances“[Title/Abstract]) AND (“physical“[Title/Abstract] OR “physical health“[Title/Abstract] OR “physical activity“[Title/Abstract] OR “physical fitness “[Title/Abstract] OR “bodily health“[Title/Abstract] OR “physiological well-being“[Title/Abstract] OR “somatic health“[Title/Abstract] OR “physical well-being“[Title/Abstract] OR “overall health“[Title/Abstract] OR “bodily fitness“[Title/Abstract]) AND (“Mental Health“[Title/Abstract] OR “psychology“[Title/Abstract] OR “psychological well-being“[Title/Abstract] OR “emotional well-being“[Title/Abstract] OR “mental well-being“[Title/Abstract] OR “psychological health“[Title/Abstract] OR “emotional health“[Title/Abstract] OR “mental well-being“[Title/Abstract] OR “psychological disorders“[Title/Abstract])	113
Web of Science (1986–2023)	TS: “Latin dance” OR “dance” OR “sports dance” OR “tango” OR “rumba” OR “cha cha cha” OR “samba” OR “bullfighting dances” OR “cowboy dances” (Topic) and “physical” OR “physical health” OR “physical activity” OR “physical fitness ” OR “bodily health” OR “physiological well-being” OR “somatic health” OR “physical well-being” OR “overall health” OR “bodily fitness” (Topic) and “Mental Health” OR “psychology” OR “psychological well-being” OR “emotional well-being” OR “mental well-being” OR “psychological health” OR “emotional health” OR “mental well-being” OR “psychological disorders” (Topic)	237

### Eligibility criteria

To find references, the PICOS model was employed. PICOS is an abbreviation for the following concepts: (1) population, (2) intervention, (3) comparison, (4) outcome, and (5) research design. Each PICOS component was utilised as a standard for inclusion in survey papers. For a study to be eligible, each of the following entry conditions shall be fulfilled: Table 3.

**Table 3:** Inclusion criteria based on PICOS circumstances

PICOS	Detailed Inclusion Criteria
Population	Irrespective of the characteristics of the population, for example, age, sex, and ethnic groups
Interventions	Latin dance
Comparisons	No Latin dance exercise group or exercise group in the control group
Outcomes	Physical and mental health performance
Study designs	RCT or Non-RCT

1. The study population had to be healthy or chronically ill but not disabled, and not have a dance background. Regardless of gender or age .
2. Latin dance training is at least 4 weeks.
3. Studies must include the effects of at least one Latin dance intervention on a person’s physical and mental health.
4. Articles must be randomized controlled trials or quasi-experimental studies.
5. Physical and/or mental health outcomes reported.
6. Studies published in English.

### Study selection

Articles that matched the inclusion criteria were chosen and included by two separate authors. This review used the EndNote 20 citation management system to eliminate duplication. Paper titles and abstracts were evaluated to decide which ones may be included in this study. In the event of a disagreement over the selection of an article, a third author was consulted to review the entire work and make a final decision.

### Data extraction and quality assessment

Following the completion of the data search, data from eligible studies were retrieved in the following formats: (1) author, title, publication year; (2) sample size, control group; (3) participant characteristics (age, sex, and so on); (4) intervention characteristics (type, duration, and frequency); (5) study design; and (6) research results. The information was abstracted into a standard form by one author, and it was checked by the other.

The PEDro scale ( www.pedro.org.au ) has been demonstrated to be a valuable measurement of experiment quality in the construction of a systematic review. The PEDro Scale is used to assess four basic approaches in a research, including random-procedure, blind technology, group comparison and data analysis. The evaluation of the 11 PEDro items was carried out by two highly trained, independent evaluators with a YES (1-point) or No (0-marks) and the dispute was solved by a third reviewer. Eligibility criteria, however, were not taken into account in the overall score, as it concerned external validity. The overall PEDro score is between 0 and 10, with a higher score reflecting a better methodology. The higher the PEDro score, the better the approach. Studies with a score of 8 to 10 were assessed as qualitatively of methodological excellence, with a grade of between five and seven being of good quality, with a rating of three to four being of moderate quality, and a rating of less than three being of low quality [29].

## Results

### Study selection

**Fig. 1:**
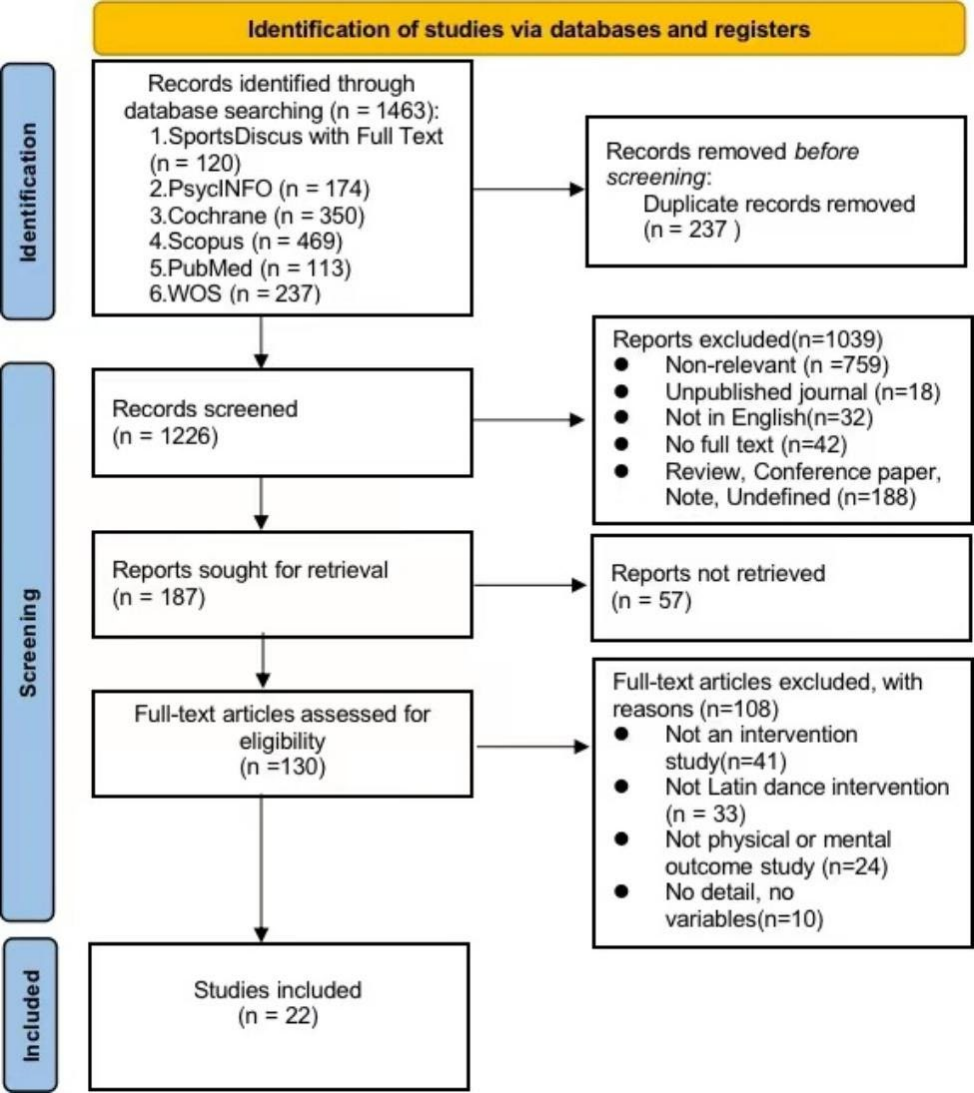
Preferred Reporting Items for Systematic Reviews and Meta-analysis (PRISMA 2020) flow diagram of the study screening process

Figure 1 illustrates the workflow for selecting records. In all, 1463 possible articles (120 from SportsDiscus with Full Text; 174 from PsycINFO; 350 from Cochrane; 469 from Scopus; 113 from PubMed; 237 from the Web of Science). After exclusion of the duplicates, the title and abstract of data were assessed for eligibility. After elimination at the title and abstract level 1039 articles, the remaining 130 articles were subsequently read. After reading, 108 other papers were excluded, and 22 related papers met the requirements of classification and were added to the qualitative summary.

### Study quality assessment

The evaluation of study quality by PEDro is shown in Table 4. The data from all investigations ranged from 3 to 7 on the PEDro scale. All studies met the criteria for eligibility, baseline comparability, and between-group comparisons. It is difficult to blind participants, assessors, and therapists because it is a dance training exercise combined with professional training and the potential of harm. Nonetheless, the studies might attempt to ensure that all patients received treatment.

**Table 4:** Scores for methodological quality evaluation are summarized

References	Eligibility criteria	Random allocation	Concealed allocation	Baseline comparability	Blind subjects	Blind therapists	Blind assessors	Adequate Follow-up	Intention to Treat Analysis	Between group comparisons	Point estimates and variability	PEDro score
Zheng,2021 [30]	1	0	0	1	0	0	0	0	0	1	0	3
Wang,2019 [31]	1	0	0	1	0	0	0	0	0	1	0	3
Soltero,2020 [9]	1	0	0	1	0	0	0	0	0	1	1	4
Sofianidis,2017 [14]	1	1	0	1	0	0	0	0	0	1	1	5
Romero,2012 [32]	1	1	0	1	0	0	0	0	0	1	1	5
Rios,2015 [33]	1	1	0	1	0	0	0	1	1	1	1	7
Qi,2021 [34]	1	0	0	1	0	0	0	0	0	1	1	4
McKinley,2008 [35]	1	1	1	1	0	0	0	0	0	1	1	6
Marquez,2017 [26]	1	1	0	1	0	0	0	0	0	1	1	5
Marquez,2014 [36]	1	0	0	1	0	0	0	0	0	1	0	3
Marquez,2022 [17]	1	1	0	1	0	0	0	0	0	1	1	5
Mangeri,2014 [37]	1	0	0	1	0	0	0	0	0	1	1	4
Li,2022 [38]	1	1	0	1	0	0	0	0	0	1	1	5
Huang,2022 [39]	1	0	0	1	0	0	0	0	0	1	1	4
Hackney,2007 [40]	1	1	0	1	0	0	1	0	0	1	1	6
Duberg,2020 [41]	1	1	1	1	0	0	0	0	0	1	1	6
Domene,2016 [21]	1	0	0	1	0	0	0	0	0	1	1	4
Banio, 2020 [42]	1	0	0	1	0	0	0	0	0	1	1	4
Ambegaonkar,2022 [43]	1	1	0	1	0	0	0	0	0	1	1	5
Aguinaga,2019 [10]	1	1	0	1	0	0	0	0	1	1	1	6
Aguinaga,2017 [44]	1	1	0	1	0	0	0	1	0	1	0	5
Adilogullari,2014 [27]	1	0	0	1	0	0	0	0	0	1	1	4

### Population characteristics

The following population characteristics of the 22 studies included in this review were assessed: (1) Sample size: a total of 1,764 subjects from 22 studies, ranging from 10 to 332; (2) Gender: Only one study had only female subjects, nine of which did not specify gender, and the rest included both male and female subjects; (3) Age: The age range was from 11 to 91. Only three studies were under 18 years old, and the other 19 studies looked at adults or college students or older adults.

### Intervention characteristics

Table 5 depicts various essential study intervention features, such as intervention type, duration, and frequency. In terms of intervention type, all studies used Latin dancing as the primary intervention strategy. Because Latin dance includes many kinds, some research only study one kind of Latin dance. Some articles refer to Latin dance as sports dance. There were three studies called Tango dance, one comparing the effects of Latin dance with tai Chi, and another comparing Latin dance with Pilates.

**Table 5:** Population, Intervention and Outcome

Study	Population	Intervention						Outcome
		Age	Sex	Type	Duration	Frequency	Study Design	
Zheng,2021 [30]	N = 200 College student	18–23	100 M 100 F	Sports dance	8 weeks	2 times/week	RCT	Psychological stress ↓. Self-satisfaction ↑
Wang,2019 [31]	N = 90 College student	20–22	unknow	Latin dance	8 weeks	2 times/week	RCT	PA, flexibility ↑
Soltero,2020 [9]	N = 20 Breast cancer survivors (BCS)	25–75	unknow	Latin dance and Tai Chi	8 weeks	unknow	RCT	body fat overall ↓
Sofianidis,2017 [14]	N = 36 older adults	65–75	10 M 26 F	Latin dance and Pilates	12 weeks	2 times/week	RCT	Balance ↑
Romero,2012 [32]	N = 73 Grade 6–9 middle school student	11–16	32 M, 41 F	Latin dance	5 weeks	2 times/week	RCT	Self-efficacy↑; social connection ↑
Rios,2015 [33]	N = 40 Parkinson’s disease.	60–65	22 M 18 F	Tango dance	12 weeks	2 times/week	RCT	Motor and non-motor manifestations↑
Qi,2021 [34]	N = 60 College student	18–23	unknow	Sports dance	10 weeks	unknow	RCT	motivation, cognition, attitude, and behavior ↑
McKinley,2008 [35]	N = 30 older adults	62–91	8 M 22 F	Tango dance	10 weeks	2 times/week	RCT	strength and walk speed ↑
Marquez,2017 [26]	N = 57 older adults	$\ge$55	unknow	Latin dance	16 weeks	2 times/week	RCT	Cognitive function↑
Marquez,2014 [36]	N = 332 older adults	$\ge$55	unknow	Latin dance	16 weeks	2 times/week	RCT	PA, self-efficacy, physical function, cognitive function ↑
Marquez,2022 [17]	N = 167 older people	$\ge$55	unknow	Latin dance	16 weeks	unknow	RCT	Physical activity (PA)↑;muscle strength ↑
Mangeri,2014 [37]	N = 100 diabetes	41–70	52 M, 48 F	Latin dance	12 weeks	2 times/week	RCT	Body weight ↓. waist circumference ↓;
Li,2022 [38]	N = 65 middle school students	11–17	24 M 41 F	Sports dance	12 weeks	2 times/week	RCT	Balance ↑
Huang,2022 [39]	N = 10 college student	19–21	5 M 5 F	Sports dance	12 weeks	2 times/week	RCT	cardiorespiratory endurance, muscular endurance, flexibility, and happiness ↑
Hackney,2007 [40]	N = 19 Parkinson’s disease.	67–75	12 M 7 F	Tango dance	13 weeks	2 times/week	RCT	addressing functional mobility deficits in individuals with PD.
Duberg,2020 [41]	N = 112 adolescent	13–18	F	Latin dance	32 weeks	2 times/week	RCT	emotional distress ↓; stress ↓
Domene,2016 [21]	N = 24	22–56	unknow	Latin dance	4 weeks	unknow	RCT	psychological distress ↓
Banio, 2020 [42]	N = 163 elderly people	$\ge$55	49 M 114 F	Latin dance	24 weeks	2 times/week	RCT	Memory ↑; mood ↑
Ambegaonkar,2022 [43]	N = 64 elderly people	$\ge$65	unknow	Latin dance	10 weeks	2 times/week	RCT	Cognitive function↑
Aguinaga,2019 [10]	N = 21 older adults	$\ge$55	5 M 16 F	Latin dance	16 weeks	2 times/week	RCT	Physical activity; Cardiorespiratory Fitness ↑
Aguinaga,2017 [44]	N = 21 older adults	$\ge$60	5 M 16 F	Latin dance	16 weeks	2 times/week	RCT	Cognition function↑
Adilogullari,2014 [27]	N = 60 college student	18–23	unknow	Latin dance	12 weeks	2 times/week	RCT	anxiety and stress ↓

*PA: Physical activity; PD: Parkinson’s Disease; RCT: Randomized Controlled Trial; M: Male; F: Female

As for the intervention duration of Latin dance, the shortest intervention duration was 4 weeks and the longest was 32 weeks. Most studies focused on intervention duration of 12 and 16 weeks. Three studies lasted eight weeks, three studies lasted 10 weeks, and only one study lasted 24 weeks.

As for the frequency of intervention, only four studies did not report frequency, and the remaining 18 studies all had an intervention frequency of twice a week.

### Outcome

This systematic review contained 22 studies that matched the inclusion criteria (see Table 5). The investigations included 1,764 people ranging in age from 11 to 91 years old. Most of the research focused on the effects of Latin dance on physical health outcomes such as cardiovascular health, strength, and flexibility. A few research, however, investigated the impact of Latin dance on mental health outcomes such as anxiety, depression and cognitive function.

#### Effect of latin dance on physical health

Table 5 shows that Latin dance intervention has an impact on the following aspects of physical health: cardiovascular health, muscle strength and tone, flexibility and balance, and weight loss. Aguiñaga and Marquez [10] show that cardiovascular health involves the whole body, and Latin dance interventions can provide excellent cardiovascular exercise, which can help improve heart health and physical activity. One study [17] of 167 older adults who received 16 weeks of Latin dance intervention training showed improved physical activity and muscle strength. The movements in Latin dance require coordination and balance, which can improve these skills over time. In Sofianidis study, a 12-week intervention in older adults using Latin dance and Pilates was found to improve balance in older adults [14]. Two studies on subjects with Parkinson’s disease, after 12 and 13 weeks [33, 40] of Latin dance intervention respectively, showed that functional mobility deficits in these subjects were significantly improved. Because Latin dance often involves stretching and movements that can improve flexibility and range of motion. Wang also conducted an eight-week study of Latin dance in college students aged 20 to 22, confirming that Latin dance can improve physical activity and physical flexibility in college students [31]. Studies have shown that Latin dancing can also help people lose weight [9, 37], Dancing is a fun way to burn calories, which can lead to weight loss and improved body composition.

#### Effect of latin dance on mental health

Table 5 shows that the Latin dance intervention had an impact on the following aspects of mental health: Stress relief; Boosts mood; Social connection; Improved cognitive function. Duberg conducted an eight-month Latin dance intervention with adolescent girls ages 13 to 18 and found that they reported reduced levels of emotional distress and stress [41]. Dancing can be a great way to relieve stress and tension, and the social aspect of Latin dance can also provide emotional support. In one study of older adults, after 24 weeks of a twice-weekly Latin dance intervention, participants’ memory and mood became more positive [42]. In a study of college students, after eight weeks of twice-weekly Latin dance interventions, participants’ psychological stress decreased and self-satisfaction increased [30]. Exercise in general is known to release endorphins, which can improve mood and decrease symptoms of depression and anxiety [45]. In a Latin dance intervention study of middle school students in grades 6 through 9, after five weeks of Latin dance twice a week, the middle school students’ self-efficacy and social connection became stronger [32]. Dancing can provide opportunities for social interaction and building friendships, which can improve overall well-being and reduce feelings of isolation. Three studies have demonstrated that Latin dance interventions can improve cognitive function. Among them, a 10-week Latin dance intervention for college students aged 18–23 showed improvements in motivation, cognitive ability, attitude and behavior [34]. The other two studies [36, 44] were Latin dance intervention for the older adults for 16 weeks, and the results both confirmed that Latin dance intervention can improve the physical function and cognitive function of the elderly. Ambegaonkar’s latest study also confirms that a 10-week, twice-weekly Latin dance intervention can effectively improve cognitive function in older adults [43]. Latin dance can improve cognitive function by challenging the brain with complex movements and sequences. This can improve memory, concentration, and overall cognitive performance.

## Discussion

The review included 22 studies, involving diverse populations and interventions. The outcomes assessed in these studies varied, but common themes emerged. Latin dance was consistently associated with positive effects on psychological well-being, including reduced psychological stress, enhanced self-satisfaction, and improved self-efficacy. Moreover, it was found to increase physical activity levels, flexibility, balance, and cognitive function. Latin dance interventions also showed promising results in improving body composition, including reducing body fat and waist circumference. The findings of this systematic review provide robust evidence supporting the positive effects of Latin dance on both physical and mental health. The results suggest that Latin dance interventions can be effective across different populations, including college students, breast cancer survivors, older adults, individuals with Parkinson’s disease, and adolescents. These findings have important implications for the development of targeted dance programs for promoting health and well-being.

### Effect of latin dance on physical health

Latin dance has emerged as a promising intervention for improving physical health outcomes, as supported by the latest research and clinical trials. This comprehensive discussion aims to examine and contextualize the findings from recent studies, shedding light on the effect of Latin dance on various aspects of physical health. By exploring the mechanisms underlying these effects and addressing the clinical implications, this discussion aims to provide valuable insights for researchers and healthcare professionals.

Cardiovascular Endurance: Recent studies have consistently demonstrated the positive impact of Latin dance on cardiovascular endurance. Aerobic dance routines, characterized by continuous rhythmic movements, have been shown to increase heart rate and oxygen consumption, thereby enhancing cardiovascular fitness [46]. These findings align with earlier research by Gabriela Cristina dos Santos [47], who reported improvements in cardiovascular endurance among participants engaged in Latin dance programs. The sustained physical activity involved in Latin dance contributes to the prevention and management of chronic diseases such as cardiovascular disease, obesity, and diabetes.

Muscular Strength and Power: Latin dance has also been found to improve muscular strength and power. The dynamic movements and weight-bearing nature of Latin dance routines stimulate the activation and development of major muscle groups. Wang demonstrated significant improvements in muscular strength and power among college students participating in an 8-week Latin dance intervention [31]. These findings align with previous studies investigating the effects of Latin dance on muscle strength and power [14, 48]. The enhanced muscular strength and power gained from Latin dance can contribute to overall physical fitness and functional capacity.

Flexibility: Flexibility is a crucial component of physical fitness, and Latin dance has been shown to positively impact flexibility. Through its varied movements and stretches, Latin dance promotes the range of motion in joints and improves flexibility [39]. Wang reported significant improvements in flexibility among college students participating in an 8-week Latin dance program. The rhythmic and flowing motions characteristic of Latin dance facilitate muscle elongation and joint mobility, thereby enhancing flexibility [31].

Balance and Coordination: Latin dance requires precise movements, coordination, and balance control. Studies have consistently demonstrated the positive effects of Latin dance on balance and coordination, particularly among older adults [14]. The complex footwork patterns and weight shifting movements involved in Latin dance routines challenge individuals to improve their proprioception and balance control. In a study of middle school students, Li demonstrated that a 12-week Latin dance intervention improved balance and coordination in middle school students [38]. These findings suggest that Latin dance can be an effective strategy for fall prevention and reducing the risk of injuries associated with impaired balance and coordination.

Body Composition: Latin dance has shown promise in influencing body composition, particularly in reducing body fat and waist circumference. Dance routines, involving continuous movement patterns and energy expenditure, can contribute to calorie expenditure and promote weight loss. Soltero [9] reported significant reductions in body fat among breast cancer survivors engaged in an 8-week Latin dance and Tai Chi intervention. Additionally, Latin dance has been associated with improvements in waist circumference, contributing to a healthier body composition [37]. These findings suggest that Latin dance can be an effective intervention for individuals aiming to achieve and maintain a healthy body weight and composition.

Overall, the discussion of recent research and clinical trials provides compelling evidence supporting the positive effects of Latin dance on physical health outcomes. Latin dance interventions have demonstrated improvements in cardiovascular endurance, muscular strength and power, flexibility, balance and coordination, as well as body composition. These findings highlight the potential of Latin dance as an enjoyable and effective form of physical activity for individuals of various age groups and health conditions. Incorporating Latin dance into clinical practice and public health campaigns can be a cost-effective strategy for promoting physical activity and preventing chronic diseases.

### Effect of latin dance on mental health

Latin dance has gained recognition as a potential intervention for improving mental health outcomes, as supported by emerging research and clinical trials [41]. This comprehensive discussion aims to examine and contextualize the findings from recent studies, shedding light on the effects of Latin dance on various aspects of mental health. By exploring the underlying mechanisms and addressing the clinical implications, this discussion aims to provide valuable insights for researchers and healthcare professionals.

Anxiety and Depression: Anxiety and depression are prevalent mental health conditions that can significantly impact individuals’ quality of life. Recent research has suggested that Latin dance can serve as a beneficial intervention for alleviating symptoms of anxiety and depression [30]. Studies have shown that Latin dance promotes the release of endorphins, natural mood-boosting substances in the brain, which can help reduce symptoms of anxiety and depression [42]. Moreover, the social and enjoyable nature of Latin dance provides individuals with a positive and engaging activity that can help combat feelings of isolation and improve overall mood [27].

Self-esteem and Body Image: Latin dance can have a positive impact on self-esteem and body image, which are closely linked to mental well-being. Engaging in Latin dance provides individuals with a sense of accomplishment and mastery, contributing to improved self-esteem. Duberg found that Latin dance interventions increased self-esteem among teenage girls [41]. Furthermore, Latin dance encourages individuals to embrace and appreciate their bodies, promoting a positive body image [36]. The rhythmic movements and self-expression inherent in Latin dance can foster a sense of body acceptance and confidence.

Stress Reduction and Emotional Well-being: Latin dance has been shown to provide a form of emotional expression and stress relief, contributing to improved emotional well-being. The combination of music, movement, and self-expression in Latin dance allows individuals to release emotions and experience a sense of catharsis. Studies have reported reductions in stress levels among individuals participating in Latin dance interventions [32]. Additionally, the enjoyment and engagement associated with Latin dance can contribute to positive emotions and overall well-being [34].

Social Connectedness: Social connectedness is an essential aspect of mental health, and Latin dance provides opportunities for social contact and involvement. Engaging in Latin dance classes or group activities fosters a sense of belonging and connection to others. Romero found that Latin dance improved social connections among young people [32]. The social interactions and support within the Latin dance community can combat loneliness and social isolation, which are risk factors for poor mental health outcomes.

Cognitive Function: Latin dance has shown potential in improving cognitive function, particularly in the domains of attention, memory, and executive function [44]. The complex choreography and coordination required in Latin dance routines challenge individuals’ cognitive abilities. Research suggests that Latin dance interventions can enhance cognitive performance and promote neuroplasticity [26, 43]. These cognitive benefits of Latin dance have significant implications for promoting healthy aging and preventing cognitive decline.

Overall, the discussion of recent research and clinical trials provides evidence supporting the positive effects of Latin dance on mental health outcomes. Latin dance interventions have shown promise in reducing symptoms of anxiety and depression, improving self-esteem and body image, reducing stress, fostering social connectedness, and enhancing cognitive function. These findings highlight the potential of Latin dance as a holistic approach to promoting mental well-being. Incorporating Latin dance into mental health interventions and wellness programs can provide individuals with a enjoyable and culturally enriching activity that supports their mental health needs. Latin dance holds promise as a valuable tool in improving mental health outcomes and enhancing overall well-being.

### Public health implications

The findings regarding the effects of Latin dance on physical and mental health have important implications for public health. Latin dance can be a valuable tool in promoting overall well-being and addressing health disparities in diverse populations. Incorporating Latin dance into public health initiatives can provide a cost-effective and accessible approach to improving population health.

One of the key public health implications of Latin dance is its potential to promote physical activity and combat sedentary lifestyles. Regular physical activity is crucial for preventing chronic diseases, such as cardiovascular disease, obesity, and diabetes [49]. Latin dance, with its energetic movements and rhythmic patterns, offers an enjoyable and engaging form of exercise that can attract individuals who may be less motivated by traditional exercise modalities. By integrating Latin dance into public health campaigns and programs, policymakers and healthcare professionals can encourage physical activity participation and contribute to the reduction of sedentary behavior in the population.

Latin dance interventions can also have a positive impact on social health and well-being. The social nature of Latin dance, with its group settings and partner interactions, fosters social connections and a sense of community. Loneliness and social isolation have been identified as significant public health concerns, as they are associated with adverse health outcomes [50]. Latin dance provides opportunities for social engagement and can serve as a platform for building social support networks and enhancing social cohesion within communities. By incorporating Latin dance into community programs and interventions, public health practitioners can address social determinants of health and promote social well-being.

Furthermore, Latin dance can contribute to cultural preservation and identity. It is an art form deeply rooted in specific cultural traditions and histories. By embracing and promoting Latin dance, public health initiatives can support cultural diversity and inclusivity. This recognition of cultural identity can empower individuals and communities, leading to improved self-esteem and a sense of belonging. Culturally sensitive interventions, such as Latin dance programs tailored to specific populations, can effectively address health disparities and reduce barriers to healthcare access among marginalized groups.

Public health strategies that integrate Latin dance should consider partnerships with community organizations, educational institutions, and healthcare providers. Collaborative efforts can help ensure the sustainability and scalability of Latin dance interventions, as well as facilitate knowledge exchange and capacity building. Additionally, rigorous evaluation studies are needed to assess the long-term effects and cost-effectiveness of Latin dance interventions in diverse settings and populations.

In conclusion, the public health implications of Latin dance are substantial. By incorporating Latin dance into public health initiatives, policymakers and healthcare professionals can promote physical activity, enhance social well-being, and support cultural diversity. Latin dance offers a unique and engaging approach to improving population health and addressing health disparities. Further research, collaboration, and program implementation are necessary to fully realize the potential of Latin dance as a public health intervention.

## Limitations and future research

This systematic review has certain limitations that should be acknowledged. Firstly, the inclusion criteria and search strategy employed may have influenced the selection of studies, potentially leading to the exclusion of relevant research. Future reviews could consider broader criteria and additional search strategies to ensure a more comprehensive representation of the literature.

Secondly, there was variation in the methodological quality and sample sizes of the included studies, which could have influenced the overall findings. Future research should aim for larger sample sizes, longer intervention durations, and standardized protocols to establish more robust evidence regarding the effects of Latin dance on mental health.

Thirdly, the generalizability of the results may be limited due to the specific populations and settings examined in the reviewed studies. Most of the included studies focused on particular groups, such as college students, older adults, or individuals with specific health conditions. Consequently, caution should be exercised when extrapolating the findings to broader populations. Future research should aim to include more diverse populations to improve the external validity of the results.

Additionally, long-term studies with extended follow-up periods are warranted to assess the sustainability of the effects and determine whether the benefits of Latin dance endure over time. Ethical considerations should be carefully addressed in future studies. This includes obtaining informed consent from participants, ensuring their safety and well-being, and maintaining confidentiality of personal information. Moreover, researchers should consider employing rigorous study designs, such as randomized controlled trials, to establish causal relationships between Latin dance interventions and health outcomes.

## Conclusion

This systematic review’s findings give considerable evidence that Latin dance has an influence on physical and mental health. According to the findings of this systematic research, Latin dance is a promising kind of physical activity. Latin dance can improve cardiovascular endurance, muscle strength, flexibility, and balance ability, and is an effective exercise for weight loss. Latin dancing can also reduce symptoms of depression and anxiety, improve cognitive function, improve mood and social connection, and reduce feelings of loneliness. Given these benefits, Latin dancing could be a useful addition to public health programs. Incorporating Latin dance into public health campaigns and initiatives can be a low-cost and accessible technique for increasing physical activity and enhancing overall health and well-being. Further research is required to better understand the causal effects of Latin dance and its potential as a method of intervention for those suffering from certain health concerns.

## Data Availability

The dataset supporting the conclusions of this article is included within the article.

## Abbreviations

PA: Physical activity
PD: Parkinson’s Disease
RCT: Randomized Controlled Trial
M: Male
F: Female

## References

[1] Purcell K, Taylor J, West K, Haynes A, Hassett L, Sherrington C. Promotion of physical activity by health professionals in a sample of six public hospitals: a cross sectional study. Health Promotion Journal of Australia 2023. 10.1002/hpja.730 37039303

[2] Lavenant P, Cacioppo M, Ansquer H, Guillaumont S, Houx L, Brochard S, Amedro P, Pons C (2023). Participation in physical activity of adolescents with congenital heart disease. Child Care Health and Development.

[3] Shah SS, Mohanty S, Karande T, Maheshwari S, Kulkarni S, Saxena A (2022). Guidelines for physical activity in children with heart disease. Ann Pediatr Cardiol.

[4] Lucibello KM, Sabiston CM, Pila E, Arbour-Nicitopoulos K (2023). An integrative model of weight stigma, body image, and physical activity in adolescents. Body Image.

[5] Jain RK, Vokes T. Physical activity as measured by accelerometer in NHANES 2005–2006 is associated with better bone density and trabecular bone score in older adults. Archives of Osteoporosis 2019, 14(1). 10.1007/s11657-019-0583-4 30826896

[6] Kim S (2023). Cognitive function, and its Relationships with Comorbidities, physical activity, and muscular strength in korean older adults. Behav Sci.

[7] Kekäläinen T, Freund AM, Sipilä S, Kokko K (2020). Cross-sectional and longitudinal Associations between Leisure Time Physical Activity, Mental Well-Being and Subjective Health in Middle Adulthood. Appl Res Qual Life.

[8] Štambuk A, Tomičić V. Experiences of older people with dancing as a form of physical activity / iskustva starijih osoba s plesom kao oblikom fizičke aktivnosti. Croatian J Educ - Hrvatski časopis za odgoj i obrazovanje 2021, 22(4).

[9] Soltero EG, Larkey LK, Kim WS, Chavez JBR, Lee RE. Latin dance and Qigong/Tai Chi effects on physical activity and body composition in breast cancer survivors: a pilot study. Complement Ther Clin Pract 2022, 47. 10.1016/j.ctcp.2022.101554 35257993

[10] Aguiñaga S, Marquez DX (2019). Impact of Latin Dance on Physical Activity, Cardiorespiratory Fitness, and sedentary behavior among Latinos attending an adult Day Center. J Aging Health.

[11] Marquez DX, Wilbur J, Hughes S, Wilson R, Buchner DM, Berbaum ML, McAuley E, Aguinaga S, Balbim GM, Vasquez PM (2022). BAILA: a Randomized Controlled Trial of Latin Dancing to increase physical activity in spanish-speaking older Latinos. Ann Behav Med.

[12] Esmail A, Vrinceanu T, Lussier M, Predovan D, Berryman N, Houle J, Karelis A, Grenier S, Minh Vu TT, Villalpando JM (2020). Effects of Dance/Movement Training vs. Aerobic Exercise Training on cognition, physical fitness and quality of life in older adults: a randomized controlled trial. J Bodyw Mov Ther.

[13] Marquez DX, Aguinaga S, Vasquez PG, Marques IG, Balbim GM, Jaldin M (2021). Dancing among older Latinos: interweaving health and culture. Latino Stud.

[14] Sofianidis G, Dimitriou AM, Hatzitaki V (2017). A comparative study of the Effects of Pilates and Latin Dance on Static and dynamic balance in older adults. J Aging Phys Act.

[15] Chan JSY, Wu JM, Deng KF, Yan JH (2020). The effectiveness of dance interventions on cognition in patients with mild cognitive impairment: a meta-analysis of randomized controlled trials. Neurosci Biobehav Rev.

[16] Shi X, Cao J (2022). FUNCTIONAL TRAINING REHABILITATION IN A LATIN DANCE INJURY. Revista Brasileira de Medicina do Esporte.

[17] Marquez DX, Wilbur J, Hughes S, Wilson R, Buchner DM, Berbaum ML, McAuley E, Aguiñaga S, Balbim GM, Vásquez PM (2022). BAILA: a Randomized Controlled Trial of Latin Dancing to increase physical activity in spanish-speaking older Latinos. Ann Behav Med.

[18] Li H, Qiu X, Yang Z, Zhang Z, Wang G, Kim Y, Kim S (2022). Effects of Cha-Cha Dance Training on the Balance ability of the healthy Elderly. Int J Environ Res Public Health.

[19] Kilic M, Nalbant SS (2022). The effect of latin dance on dynamic balance. Gait Posture.

[20] Liu YT, Lin AC, Chen SF, Shih CJ, Kuo TY, Wang FC, Lee PH, Lee AP. Superior gait performance and balance ability in latin dancers. Front Med 2022, 9. 10.3389/fmed.2022.834497 PMC9451043 36091673

[21] Domene PA, Moir HJ, Pummell E, Easton C (2016). Salsa dance and Zumba fitness: acute responses during community-based classes. J Sport Health Sci.

[22] KiliÇ M, Nalbant SS (2022). The effect of latin dance on dynamic balance. Gait Posture.

[23] Varea V. EMBODYING LATINNESS IN AUSTRALIA THROUGH DANCE. Revista Tempos e Espaços em Educação 2019, 12(31):81–96.

[24] Civil R, Lamb A, Loosmore D, Ross L, Livingstone K, Strachan F, Dick JR, Stevenson EJ, Brown MA, Witard OC. Assessment of Dietary Intake, Energy Status, and factors Associated with RED-S in vocational female ballet students. Front Nutr 2019, 5. 10.3389/fnut.2018.00136 PMC6333673 30687712

[25] Petzold MB, Bendau A, Strohle A (2020). Physical activity in the prevention and treatment of anxiety disorders. Psychotherapeut.

[26] Marquez DX, Wilson R, Aguiñaga S, Vásquez P, Fogg L, Yang Z, Wilbur J, Hughes S, Spanbauer C (2017). Regular latin dancing and Health Education May improve cognition of late middle-aged and older Latinos. J Aging Phys Act.

[27] Adilogullari I (2014). The examining the Effects of 12-Week Latin Dance Exercise on Social Physique anxiety: the Effects of 12-Week Latin Dance. Anthropologist.

[28] Page MJ, McKenzie JE, Bossuyt PM, Boutron I, Hoffmann TC, Mulrow CD, Shamseer L, Tetzlaff JM, Akl EA, Brennan SE (2021). The PRISMA 2020 statement: an updated guideline for reporting systematic reviews. Int J Surg.

[29] Albanese E, Bütikofer L, Armijo-Olivo S, Ha C, Egger M (2020). Construct validity of the Physiotherapy evidence database (PEDro) quality scale for randomized trials: item response theory and factor analyses. Res Synthesis Methods.

[30] Zheng CL, Ji HH (2021). Analysis of the intervention effect and self-satisfaction of sports dance exercise on the psychological stress of college students. Work-a J Prev Assess Rehabilitation.

[31] Wang Z, An G, Zhang W, Yang G (2019). The effect of jazz dance on physical and mental health of students with different physical fitness. J Sports Med Phys Fitness.

[32] Romero AJ (2012). A pilot test of the latin active hip hop intervention to increase physical activity among low-income mexican-american adolescents. Am J Health Promotion.

[33] Rios Romenets S, Anang J, Fereshtehnejad S-M, Pelletier A, Postuma R (2015). Tango for treatment of motor and non-motor manifestations in Parkinson’s disease: a randomized control study. Complement Ther Med.

[34] Qi J, INVESTIGATION AND ANALYSIS OF THE INFLUENCE OF SPORTS DANCE BASED ON WIRELESS NETWORK MODE ON COLLEGE STUDENTS’ MENTAL HEALTH (2021). Revista Brasileira de Medicina do Esporte.

[35] McKinley P, Jacobson A, Leroux A, Bednarczyk V, Rossignol M, Fung J (2008). Effect of a community-based Argentine Tango Dance Program on Functional Balance and confidence in older adults. J Aging Phys Act.

[36] Marquez DX, Wilbur J, Hughes SL, Berbaum ML, Wilson RS, Buchner DM, McAuley E (2014). B.A.I.L.A. — a latin dance randomized controlled trial for older spanish-speaking Latinos: Rationale, design, and methods. Contemp Clin Trials.

[37] Mangeri F, Montesi L, Forlani G, Dalle Grave R, Marchesini G: A standard ballroom and Latin dance program to improve fitness and adherence to physical activity in individuals with type 2 diabetes and in obesity. Diabetology & Metabolic Syndrome 2014, 6(1):74. 10.1186/1758-5996-6-74 PMC4082296 25045404

[38] Li L, Liu Y, Gu Y, Zhu Z (2022). Application of Heart Rate Combined with Acceleration Motion Sensor in Sports Dance Teaching. J Sens.

[39] Huang H (2022). The influence of Sports Dance on the physical and Mental Development of Contemporary College Students based on Health detection. Emerg Med Int.

[40] Hackney ME, Kantorovich S, Levin R, Earhart GM (2007). Effects of Tango on Functional mobility in Parkinson’s Disease: a preliminary study. J Neurol Phys Ther.

[41] Duberg A, Jutengren G, Hagberg L, Möller M (2020). The effects of a dance intervention on somatic symptoms and emotional distress in adolescent girls: a randomized controlled trial. J Int Med Res.

[42] Banio A (2020). The influence of Latin Dance classes on the improvement of Life Quality of Elderly People in Europe. Sustainability.

[43] Ambegaonkar JP, Matto H, Ihara ES, Tompkins C, Caswell SV, Cortes N, Davis R, Coogan SM, Fauntroy VN, Glass E (2022). Dance, music, and Social Conversation Program Participation positively affects physical and Mental Health in Community-Dwelling older adults: a Randomized Controlled Trial. J Dance Med Sci.

[44] Aguiñaga S, Marquez DX: Feasibility of a Latin Dance Program for Older Latinos With Mild Cognitive Impairment. American Journal of Alzheimer’s Disease & Other Dementiasr 2017, 32(8):479–488. 10.1177/1533317517719500 PMC10852848 28683560

[45] Blumenthal JA, Rozanski A (2023). Exercise as a therapeutic modality for the prevention and treatment of depression. Prog Cardiovasc Dis.

[46] Guo L, Chen J, Yuan W. The effect of HIIT on body composition, cardiovascular fitness, psychological well-being, and executive function of overweight/obese female young adults. Front Psychol 2023, 13. 10.3389/fpsyg.2022.1095328 PMC9891140 36743598

[47] dos Santos GC, Queiroz JdN, Reischak-Oliveira Á, Rodrigues-Krause J (2021). Effects of dancing on physical activity levels of children and adolescents: a systematic review. Complement Ther Med.

[48] Wanke EM, Schreiter J, Groneberg DA, Weisser B. Muscular imbalances and balance capability in dance. J Occup Med Toxicol 2018, 13(1). 10.1186/s12995-018-0218-5 PMC6278099 30534189

[49] Bullard T, Ji M, An R, Trinh L, Mackenzie M, Mullen SP. A systematic review and meta-analysis of adherence to physical activity interventions among three chronic conditions: cancer, cardiovascular disease, and diabetes. BMC Public Health 2019, 19(1). 10.1186/s12889-019-6877-z PMC6534868 31126260

[50] Creese B, Khan Z, Henley W, O’Dwyer S, Corbett A, Vasconcelos Da Silva M, Mills K, Wright N, Testad I, Aarsland D (2021). Loneliness, physical activity, and mental health during COVID-19: a longitudinal analysis of depression and anxiety in adults over the age of 50 between 2015 and 2020. Int Psychogeriatr.

